# Electron-stimulated purification of platinum nanostructures grown via focused electron beam induced deposition

**DOI:** 10.3762/bjnano.6.94

**Published:** 2015-04-08

**Authors:** Brett B Lewis, Michael G Stanford, Jason D Fowlkes, Kevin Lester, Harald Plank, Philip D Rack

**Affiliations:** 1 Materials Science and Engineering Department, University of Tennessee, Knoxville, TN 37996, USA; 2 Nanofabrication Research Laboratory, Center for Nanophase Materials Sciences, Oak Ridge National Laboratory, Oak Ridge, TN 37381, USA; 3 Institute for Electron Microscopy and Nanoanalysis, Graz University of Technology, Steyrergasse 17, 8010 Graz, Austria

**Keywords:** beam induced processing, direct-write, electron beam induced deposition, nano

## Abstract

Platinum–carbon nanostructures deposited via electron beam induced deposition from MeCpPt(IV)Me_3_ are purified during a post-deposition electron exposure treatment in a localized oxygen ambient at room temperature. Time-dependent studies demonstrate that the process occurs from the top–down. Electron beam energy and current studies demonstrate that the process is controlled by a confluence of the electron energy loss and oxygen concentration. Furthermore, the experimental results are modeled as a 2nd order reaction which is dependent on both the electron energy loss density and the oxygen concentration. In addition to purification, the post-deposition electron stimulated oxygen purification process enhances the resolution of the EBID process due to the isotropic carbon removal from the as-deposited materials which produces high-fidelity shape retention.

## Introduction

Focused electron beam induced deposition (FEBID) is an attractive nanotechnology application because of its unique processing latitude and high precision resolution. FEBID uses an electron beam scanned in a specific pattern to dissociate and condense precursor material onto a substrate with high shape fidelity and a high degree of flexibility in the final form of the structure [1–3]. Additionally, FEBID is a more gentle technique as compared to similar techniques (e.g., ion beam induced deposition (IBID)) which is beneficial for many applications. The major drawback to FEBID is the purity of the final deposits which results from unwanted precursor fragments left after dissociation. The immediate potential for high impact applications makes the purification strategies for FEBID an important area of study. Several strategies have been investigated for purifying EBID deposits (see Botman et al. [1] for a review). In situ purification strategies include: 1) precursors which easily decompose, for instance: WF_6_ [4], Co_2_(CO)_8_ [5], and AuClPF_3_ [6]; 2) mixed gas chemistries which react with the typically organic fragments [7–8] and 3) in situ substrate [9–10] or pulsed laser heating [11–13]. Several ex situ strategies have also been explored. Post annealing treatments in various ambients have been shown to improve the purity in PtC*_x_* deposits from the MeCpPt(IV)Me_3_ precursor and AuC*_x_* deposits from the Me_2_Au(acac) precursor [14]. Recently, pulsed laser annealing of PtC*_x_* deposits in O_2_ was also successful in fully photo-thermally inducing a C–O reaction to remove the carbon matrix and densify the platinum [15]. Electron stimulated carbon reduction in vacuum was also demonstrated for PtC*_x_* deposits [16–17]. Recently, Huth et al. explored a pulsed heating process in O_2_ which accelerates the carbon removal relative to a constant heat source and is suggested to be facilitated via a catalytic Pt–O reaction [18]. Finally, Mackus et al. have demonstrated that EBID deposited seed layers can be used as catalyst sites for selective area atomic layer deposition growth of Pt layers [19].

To this end, we have recently studied the post-deposition purification of platinum–carbon nanostructures deposited from MeCpPt(IV)Me_3_ via an electron stimulated reaction with oxygen gas [16] and water vapor [20]. Additionally we have investigated the purification of ruthenium–carbon nanostructures deposited from the bis(ethylcyclopentyldienyl) ruthenium(II) precursor via electron stimulated reaction with O_2_ [21]. The electron-stimulated H_2_O study was performed in a variable pressure scanning electron microscope (SEM) with a much higher pressure range of (10–100 Pa). In this regime, the purification appears to be reaction-rate-limited as various processing conditions could be reduced to a linear change in carbon content versus electron dose. Interestingly cross-sectional TEM studies revealed that the process occurred bottom-up where the purification rate is fastest at the end of the electron-beam range in the PtC*_x_* deposit and eventually propagates to the surface. Our previous electron-stimulated purification study in O_2_ was performed in a standard high-vacuum SEM and the O_2_ was injected with a localized gas injection system. In the previous study we examined the purification rate as a function of deposit thickness, localized oxygen pressure and oxygen temperature. The results suggested that the rate-limiting mechanism is the electron stimulated reaction of oxygen molecules adsorbed/permeated into the PtC*_x_* matrix. In this contribution we have expanded our initial study of the electron-stimulated purification in O_2_, namely we have: 1) extended our temperature study to room temperature; 2) demonstrated that the process propagates top–down; 3) studied variable current and scanning parameters; 4) compared the purification rate as a function of beam energy; 5) compared the purification of pseudo-1-dimensional wires; and 6) introduced an adsorption/permeation and reaction model, which can mimic the purification rates observed in the different regimes studied.

## Results and Discussion

### Purification rates

In our previous work [16] we demonstrated that both the purification rate and final purity increased with decreasing temperature as the oxygen gas temperature decreased between 78 and 50 °C. It was speculated that this trend could be extended to room temperature due to the increased residence time of O_2_ on the surface of the deposit, however, at temperatures below 50 °C, competitive MeCpPt(IV)Me_3_ adsorption arrested the purification process. For this study, the GIS was cleaned of all trace MeCpPt(IV)Me_3_ to mitigate the precursor contamination.

**Figure 1:**
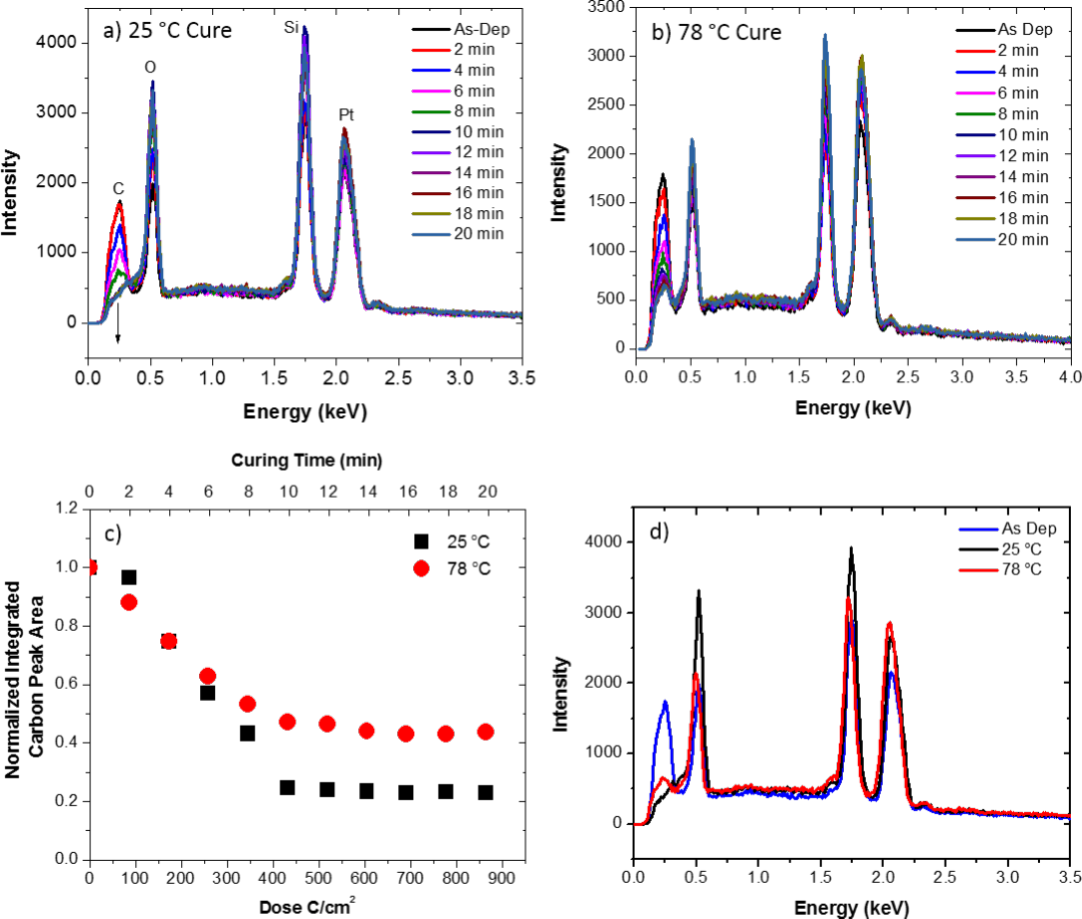
In situ EDS spectra of deposits purified using an electron beam with an energy of 5 keV, a current of 1.6 nA, a dwell time of 100 ns, and a pixel spacing of roughly 0.65 nm plotted for different purification times for the (a) 25 and (b) 78 °C O_2_ flow studies. (c) Shows the normalized integrated Pt/C ratios as a function of purification time (top axis) and estimated received dose (bottom axis). (d) Shows the final EDS spectra after 20 min of purification.

Thus, extending the study to room temperature, we re-examined the temperature dependence of the purification. Figure 1a,b illustrates in situ EDS spectra as a function of the purification time for the 25 and 78 °C studies, respectively. The initial deposit thickness before purification was ca. 100 nm. Figure 1c shows the integrated Pt/C ratios as a function of purification time and Figure 1d compares the ex situ EDS spectra for the purified deposits to the as-deposited spectra. Figure 1 clearly illustrates that the purification extent and rate is increased down to room temperature. This is consistent with our proposed mass-transport limited regime where the O_2_ residence time increases the available O_2_ on the surface (and ultimately diffused to the PtC*_x_* matrix) so subsequent electron stimulated reactions and CO*_x_* removal can proceed.

While the initial purification rates appear to be comparable, non-intuitively, the reaction rate at longer purification times and ultimately the final purity are both higher using a lower temperature reactive gas. Since higher temperature is expected to enhance oxygen diffusion, we speculate that the observed purification enhancement is due to an enhanced residence time for O_2_ at lower temperature; subsequently setting up a larger concentration gradient to enhance the diffusive transport necessary for the reaction front to propagate. It is also worth commenting on the slight peak that appears in the EDS spectra of the fully purified deposit. This peak is present because the Pt-N peak overlaps with the C-K peak. For pure platinum, the N peak to M peak ratio is about 0.09 and thus is always present even in the absence of carbon.

### Top–down reaction process

**Figure 2:**
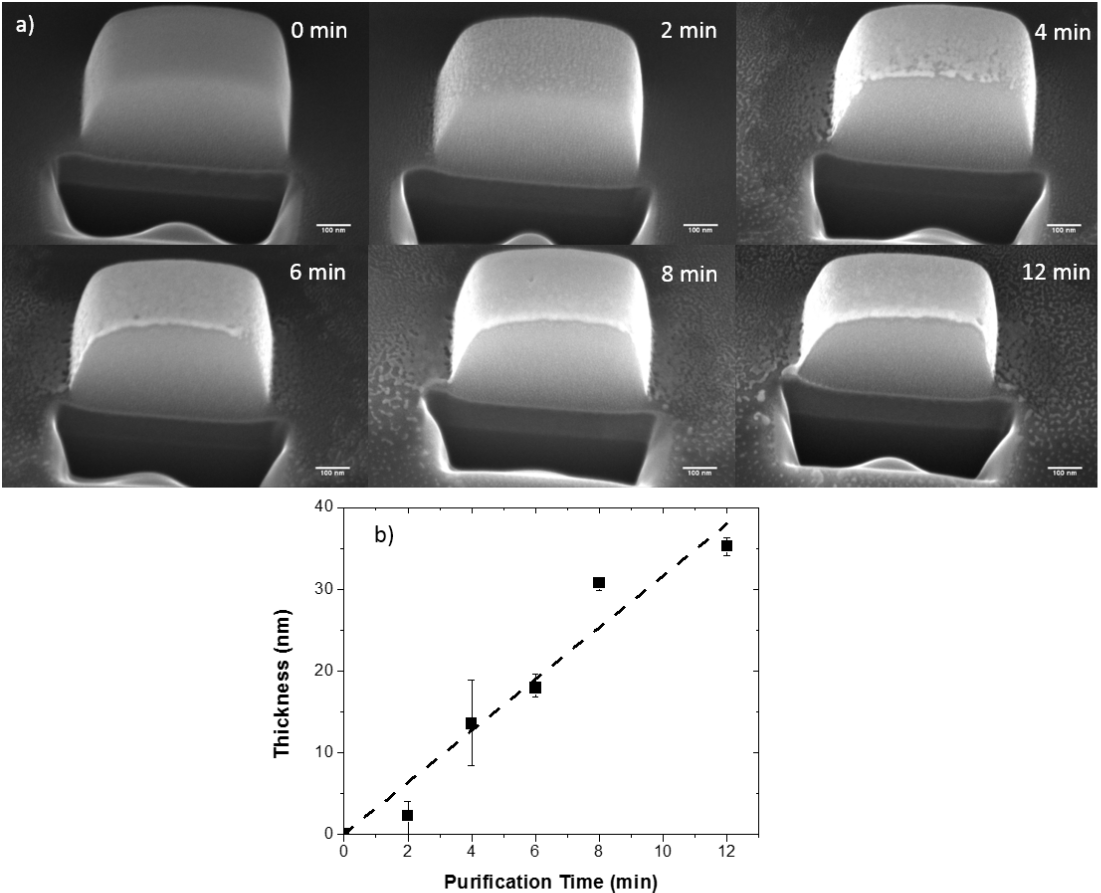
a) Cross-section SEM images of 400 nm thick PtC_x_ deposits that were purified for 1, 2, 4, 6, 8, and 12 min (reading from left to right) demonstrating the linear, top–down nature of the purification process and b) is a plot of the purified layer thickness on the top of the pad against the total purification time.

Figure 2 shows a series of relatively thick PtC*_x_* pads that were originally grown to a thickness of ca. 400 nm and subsequently purified at 25 °C at various times from 1 to 12 min. After curing, the pads were sectioned using gallium focused ion beam milling to reveal the Pt layer thickness as a function of purification time. The SEM micrographs in Figure 2 depict the bright purified platinum on top of the darker un-purified PtC*_x_* at the various purification time increments. As demonstrated, the electron stimulated O_2_ purification process appears to be occurring in a top–down manner which is distinctly different from what is observed for higher pressure H_2_O purification [20]. Consistent with the energy dispersive X-ray spectroscopy (EDS) measurements, the measured thickness versus time (Figure 2b) reveals an approximately linear purification rate (R^2^ value of 0.97). The thickness of the pad after 2 and 4 min of purification shown on the plot is included for completeness, but the error bars based on multiple measurements are quite large because the the purified layer is very thin. This suggests the O_2_ reactant surface concentration is relatively constant at the growth front. Based on amorphous carbon O_2_ etching studies by Hopf et al. [22] we previously suggested that two types of O_2_ species could be contributing to the process – namely: 1) O_2_ adsorbing simultaneous with the electron flux and 2) O_2_ adsorbed to high-binding energy carbon sites which result from electron irradiation. As the process is now revealed to occur top–down, we suggest that the process is likely facilitated via a catalytic O_2_-Pt dissociative adsorption process [18,23]; specifically, whereas O_2_ has a very low adsorption energy (and thus short residence time) on amorphous carbon the Pt surface promotes a dissociative adsorption process with a higher binding energy with consequently higher equilibrium surface coverage.

### Beam parameter studies

**Figure 3:**
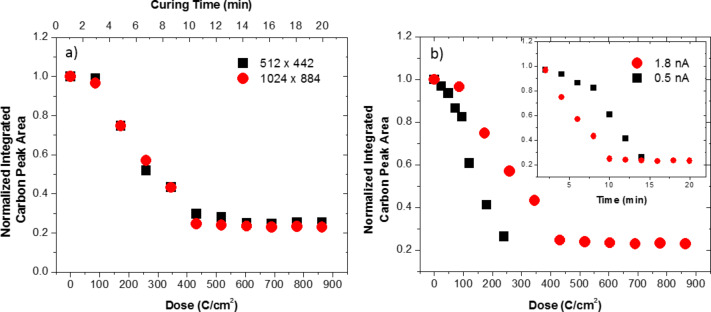
Normalized carbon peak area plotted as a function of a) curing time for the indicated pixel resolutions (note higher pixel resolution resulted in longer frame time) and b) dose for indicated beam currents. The inset depicts the same normalized carbon peak area as a function of time. The 1024 × 884 pixel resolution corresponds to ca. 0.65 nm point pitch while the 512 × 442 resolution corresponds to a point pitch of about 1.2 nm.

To explore this growth mechanism further we performed two additional studies: 1) increased pixel spacing on the live scan imaging during purification by a factor of 1.85, and 2) reduced the current to about 0.5 nA (ca. 3.6× reduction). Figure 3a compares the integrated carbon peak as a function of exposure time for the different scanning conditions. Clearly, changing the pixel spacing does not affect the purification process. The effect of increasing the pixel spacing has the effect of reducing the loop or frame time by a factor of about 3.5 (from 79 to 22 ms) and thus increasing the total number of loops by the same factor for a constant purification time. Therefore, if the process is limited by the oxygen concentration as we suspect, the local oxygen concentration in the reaction zone appears to scale with the loop time and is not saturating. This is consistent with the estimated O_2_ flux [16] of ca. 1 × 10^16^ O_2_/(cm^2^·s) and sticking probability of 0.05 approximating a monolayer coverage time on the order of 100 ms. In the second study the current was decreased with the other scanning parameters held constant. Figure 3b is a plot of the integrated carbon peak as a function of effective dose (with an inset plotted versus time). Interestingly, while the current was decreased a factor of about 3.6, the purification rate only decreased a factor of approximately two (see inset). This result implies that, at lower current, purification is more efficient (note that low current purifies at lower dose). This is consistent with a 2nd order reaction in which the reaction is a function of the electron flux (or as we will demonstrate the electron energy loss density) and the concentration of the O_2_ reactant. At the beginning of the beam dwell the per-electron purification reaction probability is highest since the O_2_ interfacial concentration is the highest. As the dwell time persists, O_2_ is consumed and the reaction probability dynamically decreases. The lower current study indicates that the integrated efficiency during the entire pixel dwell time is approximately twice as high at low current as at high current due to the dynamic consumption of the O_2_.

**Figure 4:**
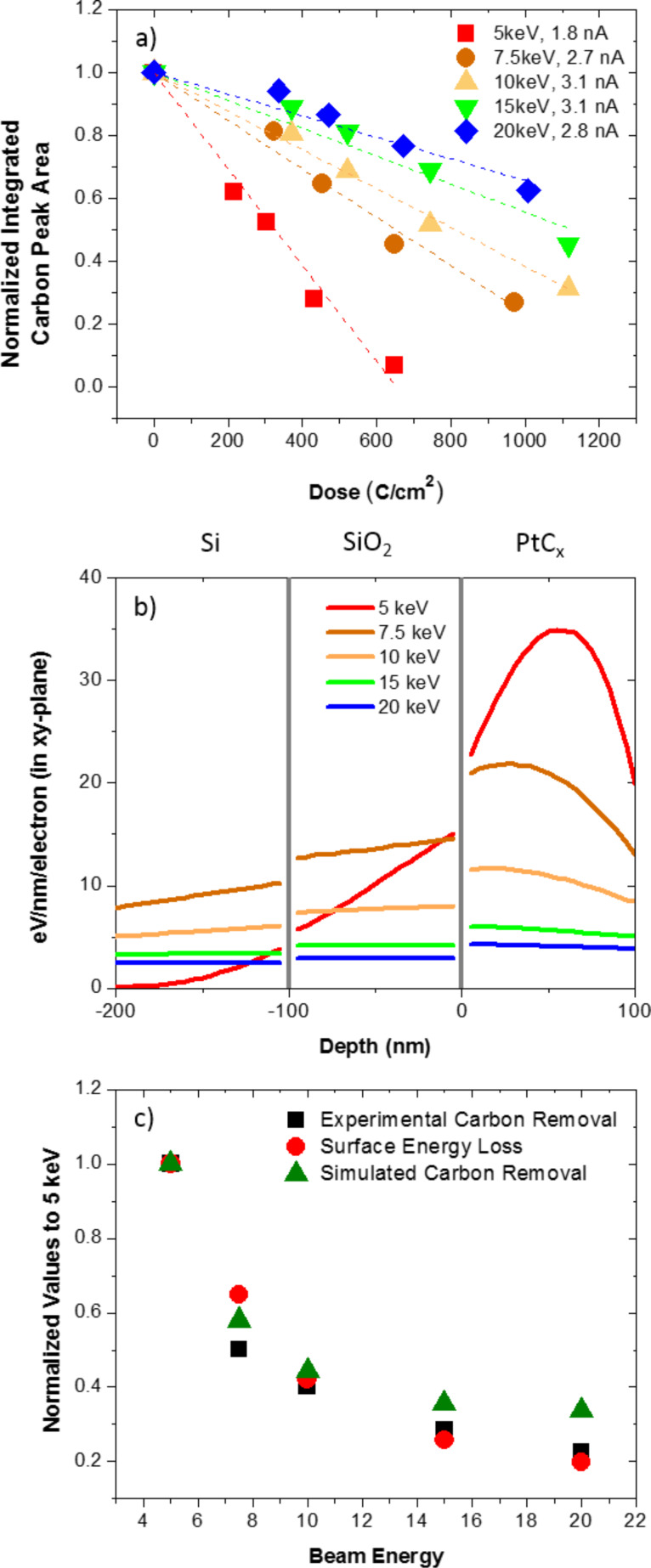
a) Normalized carbon peak area plotted as a function of dose for the beam energies shown. b) Monte Carlo simulation data depicting the electron energy loss over a 100 nm PtC_5_ film on top of 100 nm SiO_2_ as a function of *z* height (thickness) at different beam energies. The gray vertical lines represent the Si–SiO_2_ and SiO_2_–PtC_5_ interfaces. c) Comparison of the empirical purification rate to the simulated surface electron energy loss as normalized to the values at 5 keV.

To investigate the effect of the purification rate with beam energy, twenty one identical 500 × 500 nm, ca. 80 nm thick pads were grown. The beam energies studied (and associated beam current as measured in a Faraday cup) were: 5 (1.8 nA), 7.5 (2.7 nA), 10 (3.1 nA), 15 (3.1 nA) and 20 keV (2.7 nA). The temporal evolution of the purification process was investigated with purification exposure times of 5, 7, 10 and 15 min for each beam energy. Figure 4a is a plot of the integrated EDS carbon intensity as a function of exposure dose (time × current / cross-sectional area) for the different beam energies. Consistent with electron stimulated reactions, the apparent reaction cross-section decreases with increasing beam energy [24]. Because the reaction cross section is envisioned to scale with the electron energy loss in the PtC*_x_* layer, the electron energy loss was simulated at the various beam energies in 100 nm PtC_5_ films on a 100 nm SiO_2_ layer on a silicon substrate (Figure 4b). As shown, the Monte Carlo energy loss simulations in the PtC_5_ layer decreases with increasing beam energy. Since the O_2_ concentration is believed to be localized near the Pt–PtC*_x_* interface due to limited diffusion and trapping at Pt nanoparticles and the purification front, we compare in Figure 4c the normalized purification rate (normalized to 5 keV and adjusted for different currents) from Figure 4a and the near surface energy loss in the PtC_5_ layer in Figure 4b as a function of beam energy. Also shown is the normalized purification rate from our adsorption/permeation and electron stimulated reaction model as will be overviewed below. As demonstrated, good agreement between the experimental and simulated plots is observed, supporting the top–down purification model.

### Enhanced resolution

**Figure 5:**
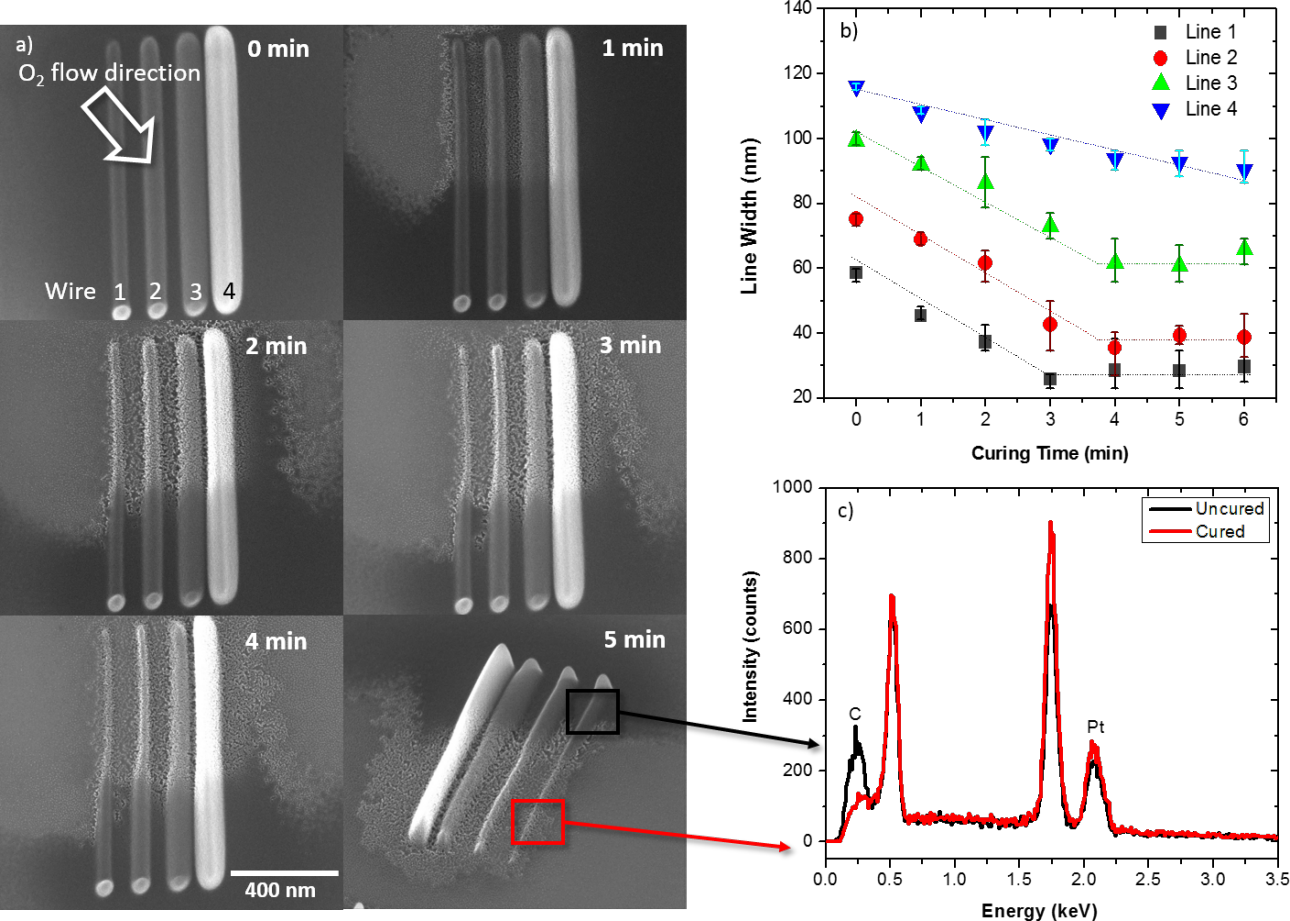
a) Images of wire deposits with different initial thicknesses (wires 1–4) purified at 1 min intervals for 6 min (inset on image). b) Estimated wire-width as a function of purification time for wires. c) EDS measurements of the purified and as-deposited part of the wire pattern.

The volumetric reduction in the PtC*_x_* deposits is proportional to the removal of carbonaceous byproduct and as demonstrated previously, purification of PtC_5_ to pure Pt results in an approx. 70% volumetric reduction [16]. Volumetric reduction in a typical 3-dimensional deposit varies in magnitude depending on the length scale in each direction. For instance, the pseudo 2-dimensional pads have asymmetric length scales as the height (ca. 100 nm) is much smaller than the lateral dimensions (500 nm); so the volume loss is dominated in the *z*-dimension with very little lateral contraction. To compare, we deposited wire patterns that were grown with progressively higher number of electron passes to investigate the purification direction and volumetric contraction of wires that are nearly symmetric in height and width. Figure 5a illustrates the purification progression of the deposits as a function of purification time; for convenience and easy comparison, we only purified half of the wires. Figure 5b summarizes the reduction in wire-width as a function of purification time for the wires grown with progressively more passes and result in different initial thicknesses (ca. 55, 90, 110, and 160 nm) and due to proximity effects [25] also increased widths (ca. 60, 75, 100, and 115 nm), respectively. Once fully purified, the wire-width reduction ceases with further irradiation time. For the wire purification of the smallest two wires one can envision an almost semi-circular cross section where each wire has a similar purification rate (slope of the wire-width versus time). The tilted image reveals that the purification is occurring in a nearly isotropic manner (both lateral and vertical contraction) and thus the complete purification time is dependent on the initial wire thickness/width. For the first and second wires there is greater than 50% wire-width reduction after a short purification time. Assuming a semi-circular cross-sectional shape and negligible relative shrinkage along the long axis, the volumetric reduction was found to be ca. 75% for wires one and two. For the thicker wires (three and four) the wire symmetry was different and thus the volumetric contraction was distributed differently across the width of the wires and the thickness of the wires. For these, volumetric reduction can be approximated more accurately by assuming a rectangular cross-section. Assuming this geometry and the final wire-widths, the volumetric reduction for both wires three and four was estimated to be 71%. This result indicates that the final shape of the deposition after purification can be predicted analytically. The overall contraction is proportional to the volumetric reduction resulting from the carbon removal from the deposit (ca. 70%) and therefore the final shape will shrink in proportion to the original dimensions.

Finally, it is worth noting the slight asymmetry or shift in the wire center for the purified wires and the residual deposition that is visible after purification. This shift is attributed to the directionality of the O_2_ gas flux; the position of the gas nozzle relative to the SEM image is noted in Figure 5a. The preferential purification is due to a slightly higher gas flux on the left side of the wires as discussed in detail elsewhere [26]. Importantly, because the O_2_-assisted electron beam purification is isotropic, one can predict the shape change due to the volumetric contraction and which fortuitously can increase the EBID spatial resolution while simultaneously purifying the deposit. The residue that becomes visible near the purified wires is a result of peripheral or proximal deposition from the electron beam tail and backscattered, type II secondary electrons and in some cases forward scattered electrons during the initial deposition [27]. This proximal deposition can be avoided with careful selection of the beam parameters during deposition. As the layer is extremely thin (a few monolayers) it can also be easily removed ex situ by with a brief focused ion beam etch.

### Microstructure

**Figure 6:**
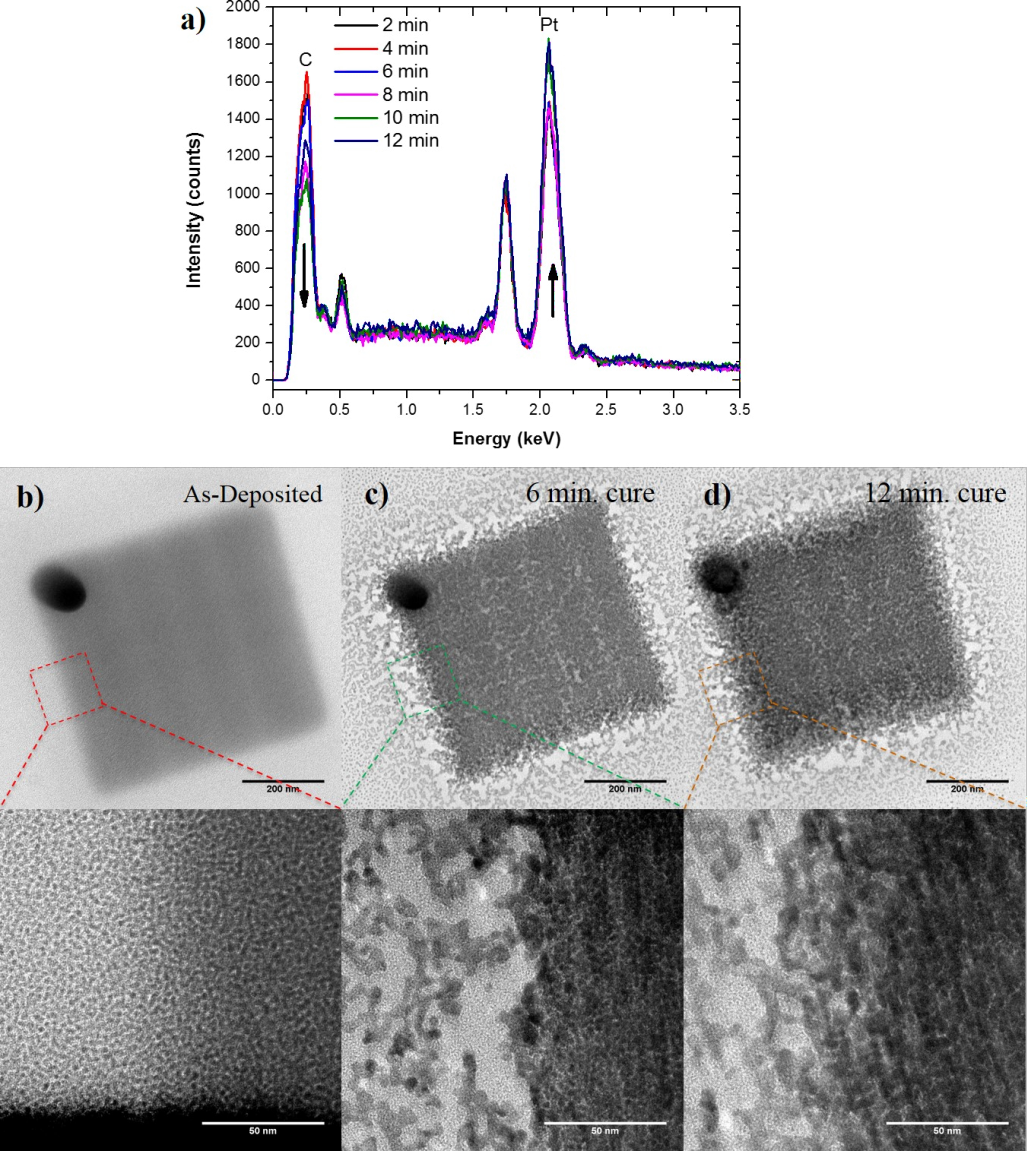
a) EDS measurements of the samples deposited and purified on the TEM membranes. TEM images of an b) as-deposited, and samples purified for c) 6 min and d) 12 min. The images reveal an increase in Pt grain size that occurs with increasing purification time.

**Figure 7:**
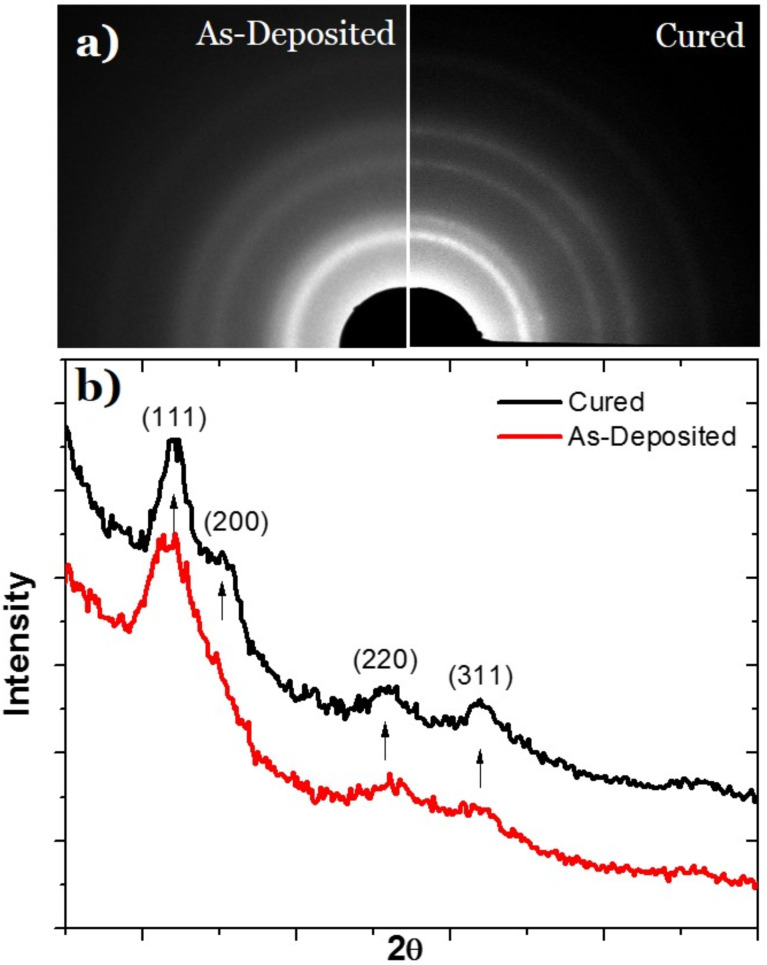
Selected area electron diffraction (SAED) patterns for O_2_ E-beam uncured (left) and cured (right) deposit. Diffraction peaks become more pronounced after curing.

Transmission electron microscopy (TEM) images of the as-deposited and cured PtC*_x_* EBID patterns were taken to compare the microstructure with progressive carbon reduction. Figure 6a illustrates initial EDS measurements of ca. 30 nm thick PtC_5_ samples that were grown on SiN*_x_* membranes confirming the decrease in carbon with increasing purification time. Interestingly, the carbon reduction is not as severe as compared to samples done on bulk substrates. While not conclusive, this could be due to two reasons: 1) an overall decrease in the energy loss in the deposits during purification due to limited backscattering from the thin membrane; 2) possible carbon deposition on the backside of the membrane due to slight outgassing from the copper tape adhesive. Figure 6b–d shows plane-view TEM images of the EBID structures at different purification times. A comparison of the TEM images reveals that the platinum grains coarsen and densify with increasing purification time. The estimated grain sizes were 1.97 nm (±0.34 nm), 3.36 nm (±0.69 nm), and 5.06 nm (±0.80 nm) for as-deposited, 6 min, and 12 min purification patterns, respectively. In order to further characterize the microstructure development during purification, selected area electron diffraction patterns (SAED) were taken for an as-deposited and purified PtC*_x_* deposit as shown in Figure 7a. Figure 7b compares the radially averaged diffraction patterns which clearly illustrates that the electron-beam assisted O_2_ purification successfully causes Pt grain densification and coarsening as indicated by the increased diffraction peak intensity and narrowing of the peak widths. The as-deposited diffraction pattern exhibits broad diffraction rings characteristic of small grain size and possibly disorder due to high carbon content. The diffraction peaks narrow as grain size increases after curing. Grain coarsening is commonly associated with an increase in electrical conductivity of the PtC*_x_* deposit as the tunnel coupling strengths increase for percolating networks [17]. For fully purified materials, our previous work [16] demonstrated a purely metallic material and thus low resistivity only one order of magnitude higher than bulk Pt. Future work will correlate purification time to the resultant percolating network and ultimately their temperature dependent electrical behavior which can reveal the granular properties of the evolving nanostructure as well as the insulator to metal transition.

### Modeling

Finally, to emulate the purification reaction we have developed a model which incorporates the O_2_ 1) surface adsorption, 2) permeation/diffusion, 3) preferential O_2_–Pt adsorption, 4) electron stimulated reaction, and 5) subsequent CO*_x_* out-diffusion. Purification experiments were simulated using a hybrid numerical approximation consisting of (1) a Monte Carlo electron scattering simulation coupled with (2) an explicit finite difference treatment of oxygen diffusion and the (3) Huen–Euler method to approximate the dissociative chemisorption of atomic oxygen on metal nanoparticle surfaces internal to the deposited solid. Electron energy loss converts bound oxygen into an activated form that is assumed to instantaneously oxidize amorphous carbon. The resulting CO*_x_* is liberated from the deposit via subsequent diffusion to the surface. The details of the simulation will be provided in a future publication and only a brief summary is provided here.

The Monte Carlo simulation is executed in order to accumulate the 3D spatial profile of the inelastic energy deposited in a semi–infinite thin film stack consisting of a PtC*_x_*_(_*_z_*_)_ deposit of thickness (*h*_film_) resting on a substrate. The 3D inelastic energy profile is then scanned across the deposit surface (to mimic the experimental beam scanning procedure) and the energy is accumulated at the center of the scanning pattern (*x* = 0, *y* = 0) in order to emulate the transiently evolving inelastic energy profile with depth (*z*-coordinate) during real experiments at the pad center. Thus, the critical parameter in the Monte Carlo simulation is the rate of electron energy loss, per unit length scattering, to the film [1–3];

[1]
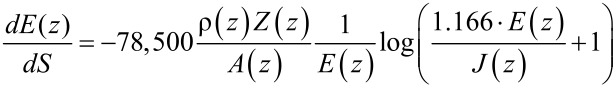


where *E* is the electron energy, *S* is the spatial path length for the scattering electron in the current voxel, ρ is the material density, *Z* is the atomic number, *A* is the atomic weight and *J* is the mean ionization potential. This value is converted into a deposited energy “concentration” with units of, e.g., [eV/nm^3^·s] for input into the transport simulation. Lastly, the semi–infinite deposit composition in the depth dimension is updated based on the transport calculations (described below) in order to account for the experimentally observed deposit densification – ρ(*z*), *Z*(*z*) and *A*(*z*) are recalculated by volume averaging based on PtC*_x_*_(_*_z_*_)_ for the next Monte Carlo simulation iteration.

In the transport simulation, O_2_ is introduced into the deposit based on a surface impingement rate derived from the input pressure *P*. Dissolution at the surface is treated according to Henry’s law *S* = *K*_eq_*P* where *S* is the solubility of oxygen and *K*_eq_ is the solubility constant. The use of this approximation requires a description of the deposit composition model. The model deposit consists of metal nanoparticles with a defined density ρ_np_ and a radius *r*_np_ distributed in an amorphous carbon matrix (*a*C) which was constructed based on TEM images of real structures. Further, the *a*C was modeled as “polymer-like” in the simulation. O_2_ dissolved and diffused within a partitioned pixel fraction based on the mean pixel composition PtC*_x_*_(_*_z_*_)_.

The transport equation treating the diffusion of the mobile O_2_ concentration (*C*_O2_*^m^*) is shown in Equation 2 as the first term on the right hand side with the remaining terms describing the interaction of mobile oxygen gas with Pt nanoparticles. An explicit finite differencing scheme is used to calculate the diffusion term in Equation 2. Importantly, the numerical approximation was derived including a variable pixel size which made it possible to “contract” the deposit based on the amount of carbon lost.

[2]
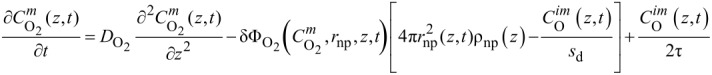


The second term in Equation 2 describes the chemisorption of mobile oxygen gas as adsorbed atomic oxygen at nanoparticle surfaces. Φ_O2_ is the impingement rate of mobile O_2_ on the nanoparticle surface (this parameter is derived from Monte Carlo simulations of a diffusing test particle impinging on a spherical nanoparticle and will be discussed in detail in the future publication) and depends on the concentration of mobile oxygen, the nanoparticle radius, depth into the deposit and time. Also important to the adsorption interaction is the sticking parameter (δ), the number of binding sites per unit nanoparticle area (*s*_d_) and the mean residence time of atomic oxygen on the nanoparticles (τ). The later appears in the third term which describes the associative desorption of atomic oxygen to form dissolved, mobile molecular oxygen. Terms 2 and 3 are combined and solved by using the improved Euler, or Heun–Euler method, applicable for a first-order differential equation which is a predictor–corrector method and is second order accurate with a cubed truncation error.

Adsorbed, immobile atomic oxygen *C*_O_*^im^* is available for electron-induced dissociation as represented by Equation 3 and provides the coupling between the Monte Carlo electron scattering simulation and the transport calculations. Specifically, term 1 on the right hand side of the equation treats the electron-driven activation of atomic oxygen using the law of mass action and treats the reaction as 2nd order (1st order in each concentration) where the units of the reaction constant are, e.g., [nm^3^/eV·s], and *C*_eV_ is the “concentration” of electron energy loss.

[3]
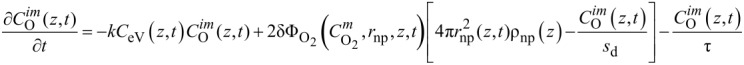


Importantly, once the oxygen is chemically “activated” the assumption is made that this species (O*) instantly reacts with *a*C yielding CO_1.5_ (without actual knowledge of the ratio of CO and CO_2_ byproduct yields, parity was assumed);

[4]
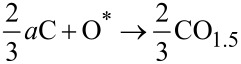


[5]
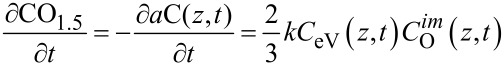


Subsequently, the liberated CO_1.5_ diffuses within the *a*C matrix and is vaporized at the deposit surface. Pixels shrink according to the amount of *a*C liberated. Conversely, the flow of both O_2_ and CO_1.5_ are prohibited at the buried deposit–substrate interface under a no–flow boundary condition. It is important to note that the spontaneous oxidation of carbon by chemisorbed atomic oxygen is neglected in the current model. As a result, the simulation results reported are expected to overestimate the rate of beam induced oxidation.

The summarized simulation flow above represents one loop of purification. Following the completion of a single loop, the PtC*_x_*_(_*_z_*_)_ composition for each *xy*-planar slice in the 3D spatial domain for the Monte Carlo simulation is updated based on the contraction of the spatial nodes in the transport simulation due to the loss of *a*C. This procedure accounts for both (1) the motion of the deposit surface as well as (2) the deposit densification.

**Figure 8:**
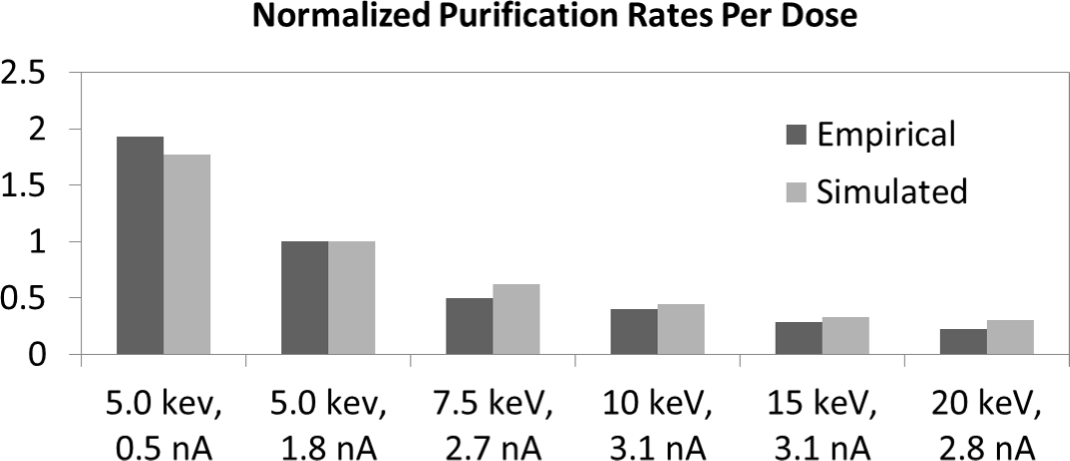
Bar graph comparing the simulated and experimental purification rates for varying beam parameters.

Table 1 summarizes of the relevant variables derived to match the 5 keV purification rate illustrated in Figure 2. Based on these variables, the different beam parameter studies were simulated. Figure 8 is a normalized (to 5 keV) bar graph of the experimental and simulated purification rates for the different beam parameters. As can be seen the purification model accurately mimics the experimental trends presented here. We will elaborate on the model and compare the O_2_ and H_2_O purification mechanisms in a near future publication.

**Table 1:** The relevant derived variables used in the simulation to obtain the correct experimentally determined purification rates.

O_2_ purification	

Electron beam energy	5 keV
Electron beam current	1800 pA

Beam scanning pixel size	0.65 nm
Beam scanning region	620 nm
Beam dwell time per pixel	100 ns

Pad thickness	100 nm
Initial pad composition (PtC*_x_*)	5 [ ]

Rate constant	4000 nm^3^/(eV·s)
Diffusion coefficient	4 µm^2^/s
Solid dissolution constant	0.03 atoms/(nm^3^·Torr)
Mean residence time (reactant on metal np)	12.5 ms
Sticking probability	0.05 [%]

Pressure (reactant gas)	2 mTorr

## Experimental

Platinum boxes were deposited onto a pre-cleaned 100 nm thermal SiO_2_ on Si substrate and then purified. The deposition and subsequent purification was performed using a FEI NOVA 600 dual-beam system equipped with FEI and Omniprobe gas injection systems. Prior to deposition, the substrate was sonicated in isopropanol for 5 min and annealed at 900 °C in an Ar-H_2_ atmosphere. The sample was then transferred to the SEM chamber and cleaned using a XEI Scientic plasma cleaner for 30 min at a pressure of 5.34 × 10^−1^ mbar. Subsequent to cleaning, the system was pumped to a base pressure of less than 3 × 10^−6^ mbar. Platinum deposition was performed using the FEI GIS and MeCpPt(IV)Me_3_ precursor which raised the chamber pressure to ≈1.5 × 10^−5^ mbar. 500 × 500 nm boxes were synthesized at this elevated pressure using the NOVA patterning software with a beam energy of 5 keV, beam current of 120 pA, point pitch of 13.55 nm, a pixel dwell time of 10 µs, and 1000 loops in order to create a pad with a thickness of about 100 nm. After deposition and pumpdown back to <3 × 10^−6^ mbar, the pads were purified using an O_2_ flow during electron beam irradiation. The oxygen flow and temperature were controlled using the Omni GIS system which was inserted to the same position as our previous work: an angle 52° with respect to the substrate surface and the lower end of the GIS nozzle at a straight line distance of 120 µm from the substrate surface. The oxygen gas line was heated to 78 °C and 25 °C though some cooling likely occurs before delivery from the nozzle. After the target oxygen temperature was reached, the oxygen was injected into the SEM chamber at a pressure of ≈1.4 × 10^−5^ mbar which was determined by controlling the valve sequence in the OmniGIS. At this point, the electron beam was used to irradiate the deposit with the simultaneous flow of O_2_. Typical purification parameters were: a beam energy and current of 5 keV and 1.8 nA, respectively, a ≈0.65 nm point pitch with a field of view of 1024 × 884 pixels (665 × 575 nm), a dwell time of 100 ns, and a typical curing time of 20 min.

To characterize the reduction in PtC*_x_* deposit size with purification, four EBID wires were deposited on a SiO_2_ substrate and purified using the same parameters as with the pads. The wire patterns were deposited with 5 keV, 28 pA beam conditions and a varying number (10,000, 15,000, 20,000 and 50,000) of EBID passes to achieve heights of 48, 70, 95, and 150 nm and widths of approximately 55 nm, 75 nm, 95 nm, and 115 nm, respectively. Half of the wire pattern’s length was purified and the other half was left in as-deposited conditions to provide a dimensional comparison to reveal shrinkage associated with purification.

To compare the resultant microstructure and grain size of progressively purified material, six samples were prepared on 30 nm thick SiN*_x_* substrates for transmission electron microscopy (TEM) imaging. The samples were deposited and purified using similar parameters reported above. The samples were purified to different times (and doses) of 2 min (51.2 C/cm^2^), 4 min (102.3 C/cm^2^), 6 min (153.5 C/cm^2^), 8 min (204.7 C/cm^2^), 10 min (255.8 C/cm^2^), and 12 min (307.0 C/cm^2^). Selected area electron diffraction was also performed to obtain diffraction patterns. These experiments were conducted in a Zeiss Libra 200 HT FE MC at 120 keV and minimal beam current to prevent morphological changes.

## Conclusion

We have studied the electron-stimulated O_2_ purification of PtC EBID deposits and have shown that the process can be extended to room temperature. Electron beam current and energy studies suggest the process is governed by a dynamic process which is a function of both the electron energy loss and oxygen concentration. Importantly, the purification front propagates from the top–down which suggests a preferential trapping or limited permeation of the O_2_ reactive gas. A model based on a 2nd order reaction rate was also demonstrated, which accurately reproduces the experimental trends. Finally, purification of pseudo-1-dimensional wires illustrate that the purification process is isotropic and conveniently the carbon reduction leads to higher resolution wires.
